# Soil solution data from Bohemian headwater catchments record atmospheric metal deposition and legacy pollution

**DOI:** 10.1007/s11356-023-25673-7

**Published:** 2023-02-08

**Authors:** Daniel A. Petrash, Pavel Krám, Katherine X. Pérez-Rivera, František Bůzek, Jan Čuřík, Frantisek Veselovský, Martin Novák

**Affiliations:** 1 Department of Environmental Geochemistry and Biogeochemistry, Czech Geological Survey, Geologická 6, 152 00 Prague 5, Czechia; 2 Institute of Soil Biology and Biogeochemistry, Biology Centre of the Czech Academy of Sciences, Na Sádkách 7, 37005 České Budějovice, Czechia; 3 Department of Biological Sciences, Virginia Tech, 926 West Campus Drive, Blacksburg, VA 24061 USA

**Keywords:** Metal pollution, Shallow response times and recovery, Vadose zone, Stable oxygen isotope, Groundwater vs. runoff contribution model, Lysimeters

## Abstract

Soil solution chemistry depends largely on mineralogy and organic matter properties of soil horizons with which they interact. Differing lithologies within a given catchment area can influence variability in soil cation exchange capacities and affect solute transport. Zero-tension and tension lysimeters were used to evaluate the fast transport of solutes in the topsoil vs. slow diffusional matrix flow at the subsoil of three contrasting lithology catchments in a mid-elevation mountain forest. Our aim was to test the feasibility of lysimeters’ hydrochemical data as a gauge for legacy subsoil pollution. Due to contrasting lithologies, atmospheric legacy pollution prevailing at the soil-regolith interface is differently yet consistently reflected by beryllium, lead, and chromium soil solution concentrations of the three catchments. Geochemical (dis)equilibrium between the soil and soil matrix water governed the hydrochemistry of the soil solutions at the time of collection, potentially contributing to decreased dissolved concentrations with increased depths at sites with higher soil pH. A complementary isotopic *δ*^18^O runoff generation model constrained potential seasonal responses and pointed to sufficiently long water-regolith interactions as to permit important seasonal contributions of groundwater enriched in chemical species to the topsoil levels. Our study also reflects subsoil equilibration with atmospheric solutes deposited at the topsoil and thus provides guidance for evaluating legacy pollution in soil profiles derived from contrasting lithology.

**Supplementary Information:**

The online version contains supplementary material available at 10.1007/s11356-023-25673-7.

## Introduction

The composition of soil solutions is altered by the deposition of solutes from the atmosphere and interactions between unsaturated soil water and shallow groundwater after regolith weathering. Additionally, seasonally variable soil water residence times and evapotranspiration can induce changes in soil solution chemistry.

The biogeochemical properties of soil solutions are largely dependent on the mineralogy and organic matter characteristics of the soil horizons with which they interact (see Brantley et al. 2007 for a revision). Differing lithologies within a given catchment area can also be reflected as variability in soil cation exchange capacities. Localized variability ensues differential weathering. For example, in a forest ecosystem with contrasting igneous bedrocks altered under the same climatic regime, it was estimated that the leaching of base cations from soils derived from mafic rocks can be up to 100-fold higher than that of soils formed after granite alteration (Krám et al. 2012; Dannhaus et al. 2018). Similarly, lithological heterogeneity regulates the mineralogy and distribution of metal reactive clays which also control elemental leaching and diffusion rates (Brantley et al. 2007).

Integrating and interpreting the multiple factors that contribute to soil solution chemistry properties—at any temporal scale—is complex. Lysimeters are used to collect soil solutions for analysis and can offer a path for investigating soil solution properties in their natural state. The study of soil solution hydrochemistry through lysimeters helps in understanding nutrient mobility and dynamics (e.g., Carey 2003; Johnson et al. 2018; Petrash et al. 2019; Makowski et al. 2020; McDowell and Potter 2022), and can be used for evaluating the prevalence of toxicants in soils (e.g., Shaheen et al. 2014; Worrall et al. 1999). Many previous studies used lysimeters for developing the understanding on pollution processes, composition, and fluxes of vadose waters (Goss et al. 2010). In this contribution, we critically evaluated the significance of elemental concentration in soil water collected using a combination of zero tension and tension lysimeters to inform legacy pollution in soil solution chemistry. For this purpose, water residence times, cation exchange properties, and the influence of differing lithologies were considered to evaluate and validate legacy pollution.

**Fig. 1:**
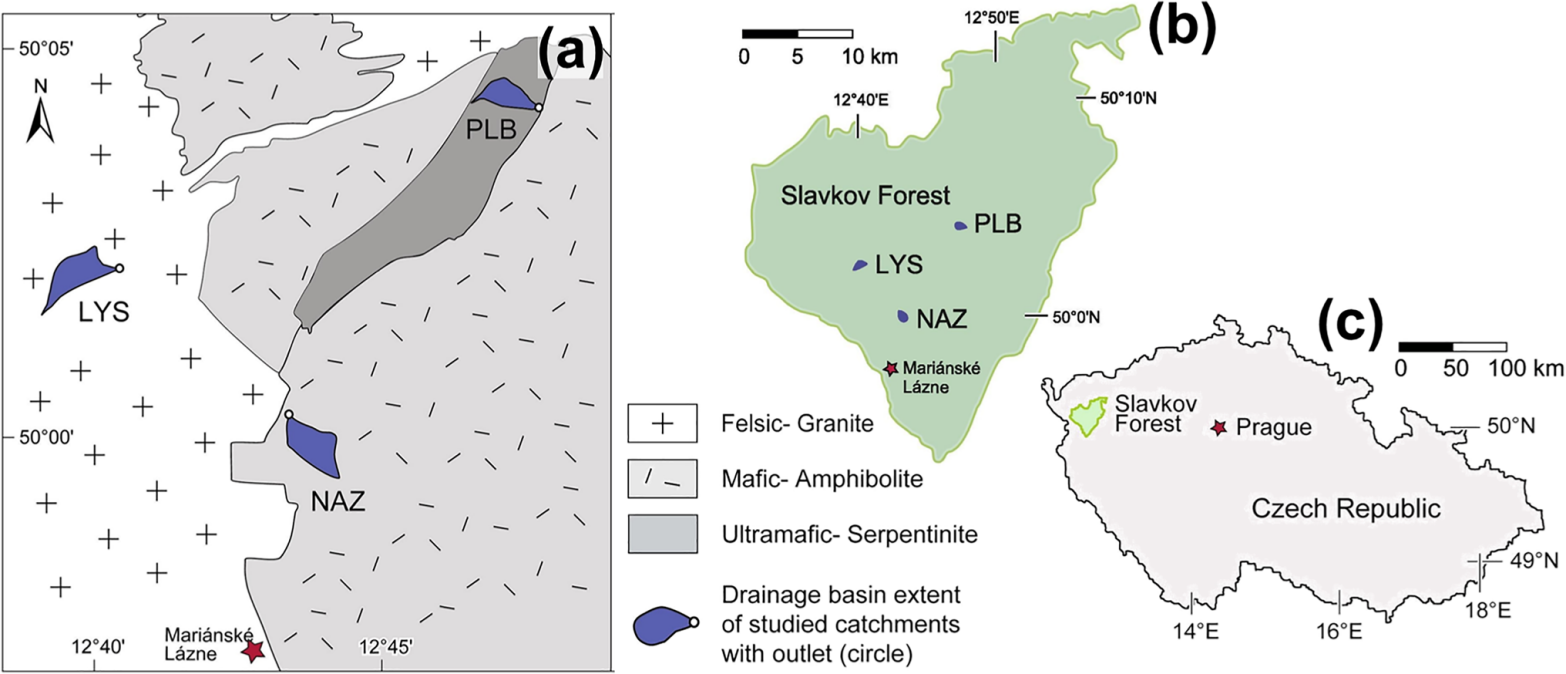
Location of the Slavkov Forest in NW Czech Republic. The discrete catchments under evaluation feature soils developed at the expense of contrasting bedrock lithologies (**a**), the location of the catchments within the Slavkov Forest (**b**), and of the forest ecosystem in NW Czechia (**c**) are also shown

Our lysimeters were placed at constant depths in soils derived from the slightly weathered (i) ultramafic, (ii) mafic, and (iii) granitic lithologies in the Slavkov Forest (Carlsbad Region, NW Czech Republic; Fig. 1). The continuously monitored sites constitute part of a temperate forest ecosystem that was heavily impacted by acid pollution linked to sulfur (S) and nitrogen (N) emissions from coal combustion during the second half of the twentieth century (Krám et al. 2012). As in other small headwater catchment areas comprising the Czech GEOMON network, the ongoing recovery of the Slavkov Forest from acidification has been long-term monitored (Helliwell et al. 2014; Oulehle et al. 2017, 2021). Across Central Europe, and notably in all mid (650 to 850 m a.s.l.) to high (> 850 m a.s.l.) elevation forests of the Czech Republic, wet and dry deposition of particulate metals derived from coal-burning power plants accompanied significant soil acidification (Bohdalkova et al. 2012, 2020; Petrash et al. 2021). Harmful deposition of metal pollutants during the 1970s to the mid-1990s resulted from scant emission treatment practices in lignite-based power and heat generation plants widespread distributed in a formerly extremely polluted area: the so-called Black Triangle region (Blažková 1996; Kolář et al. 2015). Soil metal pollution may persist today, thus creating a burden that could extend across diverse ecosystem compartments, and that is yet to be fully addressed.

Dissimilar soil profiles, cation exchange, and acid-neutralizing capacities in the catchment areas under consideration (Krám et al. 2012) are seen here as an analytical advantage given that beryllium (Be), chromium (Cr), and lead (Pb) should exhibit―under unperturbed conditions―a diffusional enrichment profile towards the felsic (Be, Pb), and mafic/ultramafic (Cr) regolith of the Slavkov Forest. In other words, concentration gradients of these metals in uncontaminated soils would solely depend on the bedrock lithology and would be diminished or augmented only by the intensity of weathering and topsoil development processes. While atmospheric deposition may complicate the integrated signal, inversed metal concentration gradients should emerge if legacy pollution exists.

**Fig. 2:**
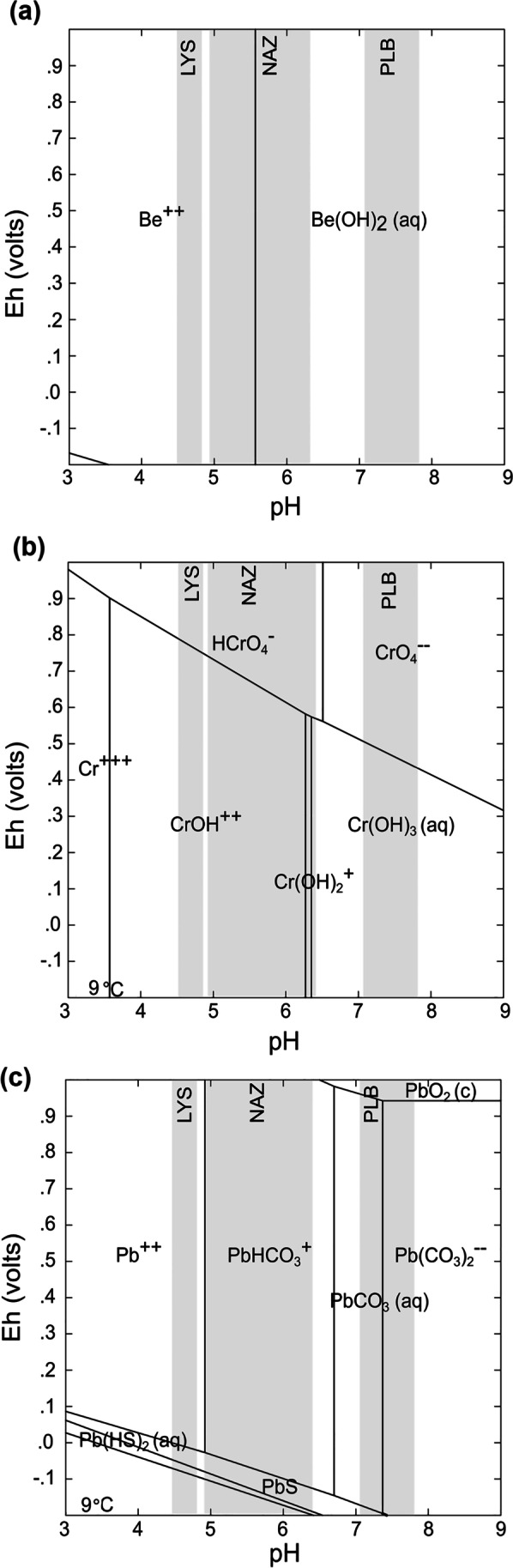
Possible thermodynamically stable phases of rain water in equilibrium with atmospheric pCO_2_ (10^−3.5^ atm) shown in Pourbaix (Eh–pH phase stability) diagrams. We depicted the pH range of the catchment areas under consideration in this study (see Fig. 1 for site acronyms). The diagrams were produced with GWB ® (community edition) using 10 ppb of the trace elements and runoff solute concentrations as reported in Krám et al. (2012, their table 4). Anionic Be.^2+^ is present in solution at pH < 5.5; in soil solutions of LYS, it therefore exists as a free ion: Be speciation is solely controlled by pH. At the pH characterizing NAZ, Be(II) is hydroxide complexed, while all Be must exist as the aqueous Be(OH)_2_ complex at PLB. The formation of Be(OH)_2_(aq) means that Be(II) can be strongly adsorbed with Al and Fe oxyhydroxides in the nearby soil systems of the study sites (**a**). Chromium cations are found in all sites chiefly in the form of aqueous complexes rather than as the free Cr(III) ions. In all sites, Cr exists as a highly mobile chromate oxyanion. Chromate could either be sorbed by iron and aluminum oxyhydroxides, particularly at the PLB and NAZ sites, or it could be mobilized as the result of seasonality (see text for discussion) (**b**). Ionic Pb has a strong affinity for bicarbonate/carbonate anions. Therefore, pH is strong control of its speciation in non-reductive soil systems. Pb carbonate complexes could form with ease in the NAZ and PLB bedrock and soils, but lead is poorly adsorbed onto oxyhydroxides. Only at LYS, Pb(II) ions may be present in soil solutions (**c**)

The selected metal pollutants listed above exhibit theoretically markedly different chemical speciation, (co)precipitation/dissolution, and adsorption/desorption behaviors (Fig. 2). We therefore hypothesize that when normalized relative to regolith base levels, lysimeter concentrations of Be, Cr, and Pb concentrations can be used as proxies for the prevalence of metal pollutants in subsoil regolith. As shallow groundwater seasonally interacts with runoff at the unsaturated zone of the soil, following such interaction topsoil lysimeters should rapidly reflect the development of measurable concentration anomalies. However, concentration anomalies may also be indicative of the infiltration of atmospheric metal fluxes. Anomalous concentrations that could unambiguously be linked to long-term runoff-shallow groundwater mixing would thus hint to deeply seated legacy Be, Cr, and/or Pb pollution. This would manifest more evidently if pore fluid-soil interactions are marked by sufficiently long mixed-source water residence times. We estimated the residence times of runoff and the contribution of groundwater to runoff recharge in the studied catchment areas by implementing a stable δ18O isotope runoff water generation model (Buzek et al. 1995; McGuire and McDonnell 2006). The isotopic data provides complimentary information on pollution legacy and remnants of toxicants within soil solution.

## Materials and methods

### Study site: the Slavkov Forest and the three-contrasting lithology, regularly monitored catchments

The Slavkov Forest is a protected forest ecosystem in the Carlsbad (Karlovy Vary) region, NW Czech Republic. It is situated between 400 and 983 m a.s.l., and in total comprises *ca.* 611 km^2^. Vegetation is dominated by a Norway spruce (*Picea abies* L. Karst.) cover that is subjected to regular harvesting. The vegetation accounts for an estimate of 101 to 277 t of biomass per ha (Table 1). Three catchments within the Slavkov Forest: (i) Lysina, LYS; (ii) Na Zeleném, NAZ; and (iii) Pluhův Bor, PLB, are environmentally monitored regularly. Their soil parental rocks encompass an igneous-metamorphic terrain that did not endure Pleistocene glacial erosion (Krám et al. 2012). The LYS, NAZ, and PLB catchments have similar elevation (above ~ 700 m a.s.l.) and climate (see below) but contrasting bedrocks. The catchments overlie felsic, mafic, and ultramafic bedrocks, respectively (Krám et al. 2017; Fig. 1). Pedodiversity in the monitored catchments correspond to their lithological variability and morphology (Table 1).

**Table 1:** Site description and related previously published data

Site ID	Coordinates	Elevation range (m a.s.l.)	Catchment area (ha)	Soil underlying lithology	IUSS soil type	Mean stream water pH (ANC)*	2015 tree biomass (t ha^−1^) ‡
LYS	50°02.2′ N, 12°39.6′ E	829–949	27	Felsic ((leuco)granite)	Podzol	4.2 (− 50)	101
NAZ	49°59.8′ N, 12°42.5′ E	736–802	55	Mafic (amphibolite)	Cambisol	6.9 (+ 210)	277
PLB	50°03.8′ N, 12°46.9′ E	690–804	22	Ultramafic (serpentinite)	Stagnosol	7.3 (+ 990)	169

^*^in μeq L^−1^, Krám et al. (2013) ‡ Krám et al. (2017)

The forest in the LYS, NAZ, and PLB catchments are characterized by humid temperate conditions (Köppen climate―type Dfc). The mean annual temperature at the catchments is about 5.9 °C, with a long-term range (1994–2019) of between − 3.1 and 15.3 °C―mean of 6.5 ± 0.1 °C (Oulehle et al. 2021). The annual sum of precipitation for the three catchments is estimated at about 1020 mm year^−1^. This value, however, is based on observations made at the LYS catchment, situated at a slightly higher altitude (Table 1). In a typical year, snow cover could be present from early November to late April.

The monitoring period evaluated here (2012–2014) spans the transition to a record 5-year drought (2014–2019) that was well-documented by the GEOMON data (e.g., Oulehle et al. 2021). During that period, contrasting stream hydrochemistry documented differences in chemical weathering among the studied catchment areas. Accordingly, low pH (mean 4.5) and a low acid neutralizing capacity (ANC) characterized the streams draining the granitic LYS, while those draining NAZ showed medium to high acid neutralizing potential, and streams draining PLB exhibited the highest measured mean pH and ANC values (Table 1).

### Soil profiling, solution sampling, and geochemical analyses

For the purpose of soil textural class description, along each of the regularly monitored catchment areas, a total of 5–6 quantitative soil pits were excavated by following methods described in Huntington et al. (1988). The location of the soil sampling pits was randomly generated while considering the forest stand age and ecological category (Chuman et al. 2021). Inside a 0.5 m^2^ reference frame, the forest floor and mineral soils were removed to a depth > 80 cm below the surface. The excavated material was first separated into the three distinct organic layers, Oi/L + Oe(F), and Oa(H). Below these layers comprised of leaves, pine needles, and twigs; a partially decomposed layer; and then the very dark layer of well-decomposed humus, respectively, the actual soil horizons were depth-defined (i.e., 0–10, 10–20, 20–40, and 40–80 cm). Mineral soil textures were analyzed by the hydrometer method ISO 11277 2009. Materials from each soil layer were weighed and sieved in the field (1 cm) and separated into cobles, soil < 1 cm, and coarse roots. Following air drying, the soil substrate was further 2-mm-sieved and sub-sampled.

Exchangeable cations in 0.1 M BaCl_2_ extracts were analyzed by atomic absorption spectrophotometry (AAnalyst Perkin Elmer 200). Exchangeable acidity was determined by titration of the extract aliquots. Cation exchange capacity (CEC) was calculated as the sum of exchangeable base cations and exchangeable acidity. The aluminum oxide content of the regolith [Al_2_O_3_] was determined by flame atomic absorption spectroscopy (AAnalyst PerkinElmer 100, Norwalk, CT) with an accuracy > 0.01 wt.% based on lab standards. Solid phase concentrations of Be, Cr, and Pb were determined by inductively coupled plasma mass spectrometry after trace-grade 8N HNO_3_ subsample digestion (Thermo Scientific X-series 2,), and with certified detection limits of 500 (Pb) and 1000 µg kg^−1^ (Cr, Be). In addition, X-ray diffraction (XRD) was used to identify clay minerals in three replicated samples of mineral soil from depths of 40 to 80 cm. The methods for XRD and analytical parameters were described by Novak et al. (2020).

A monthly hydrochemical sampling of soil solutions was performed for 3 years (March 2012–February 2014) by using a combination of zero-tension lysimeter nests installed at topsoil depths (10 to 40 cm), and Prenart tension lysimeter nests in subsoil levels. These latter lysimeters were installed at depths of 60 and 90 cm below the soil surface. Collection of soil solution samples for chemical analyses started 5 months after the installation of all lysimeters.

The methods for soil solution collection, analysis, and quality control were implemented as described in the Manual for Integrated Monitoring (ICP IM Programme Centre 1998). Zero-tension lysimeters consisted of rounded rectangular polyethylene containers with an area of 132 cm^2^. The polyethylene trays were filled with acid-washed silica sand and installed horizontally in the soil pit. Tension lysimeters were monthly pressurized to 75 MPa below atmospheric pressure (at the time of sampling) by using an electrical vacuum pump. By the end of each month, the soil solutions were collected (transferred) out from 2 L polyethylene to lysimeter collecting bottles. Soil solution pH and alkalinity were measured by titration usually 1 day after the return from the field.

After transport to the lab, the soil solution samples were refrigerated (4 °C) on acidified sample aliquots (8N trace grade HNO_3_). We determined trace metal concentrations by using the same ICP-MS instrument described above, and with detection limits of 0.02, 0.40, and 0.50 µg L^−1^ for Be, Cr, and Pb ions respectively. Electrothermal atomic absorption (AAnalyst PerkinElmer 700, Shelton, CT) was used for determining dissolved Al concentration analyses, with a detection limit of 10 µg L^−1^. On filtered un-acidified sample aliquots, Cl^−^ and SO_4_^2−^ concentrations were determined through high-pressure liquid chromatography (Knauer 1000, Germany), with detection limits of 0.30 and 0.15 mg L^−1^, respectively, and accuracy better than 20% of the reported value. Soil organic carbon (SOC) was determined by using a non-dispersive infrared CO_2_ sensor after sample ignition.

#### Lysimeter hydrochemical data treatment

In accordance with the Manual for Integrated Monitoring (op cit.), each of our nest consisted of three lysimeters. These nests produced seasonal replicates per sampling location and depth. A total of 192 observations were obtained during the whole sampling period. These observations included lysimeters nests situated at six soil profile depths (10, 20, 30, 40, 60, 90 cm) at LYS, and five lysimeters networks at depths 10, 20, 30, 60, and 90 cm in the other two sites (NAZ and PLB). The monthly soil solution hydrogeochemical observations were first considered site replicates for reducing the data to a yearly averaged, site-based dataset, and then statistically discriminated by: (i) topsoil (zero-tension) and subsoil (tension lysimeters) and (ii) season. The missing individual observations―related to, for example, low soil solution volume collection in a given month―were excluded from statistical analyses. These included descriptive statistics and factor analyses (FA).

FA is among the multivariate statistical methods more often used in hydrochemistry (Praus 2005). For FA, a correlation matrix is first generated. A correlation coefficient is rotated to maximize the relationships existing between explanatory factors and variables. This rotated correlation matrix is then used to account for the degree of mutually shared variability between individual pairs of parameters measured in our soil solutions. Eigenvalues and factor loadings for the correlation matrix are then determined. This allows for identifying correlation among groups of variables. Higher eigenvalues contribute the most to the explanatory ability of a given variable. Only two to three factors are needed to account for most of the variability in the dataset. Once the correlation matrix and eigenvalues are obtained, factor loadings are then used as a measure of the correlation between the variables and explanatory factors. This latter approach allows identifying and weighting the influence of latent factors over the variability contained in a large number of measured non-normally distributed environmental parameters (Zeng and Rasmussen 2005). The analysis was implemented in MS Excel® using the add-in code XLSTAT®. Data reduction and descriptive statistics were followed by data visualization using ggplot2 in the R statistical environment (Wickham 2016).

## Results and discussion

### Parameters potentially affecting elemental concentrations of selected pollution proxies

**Fig. 3:**
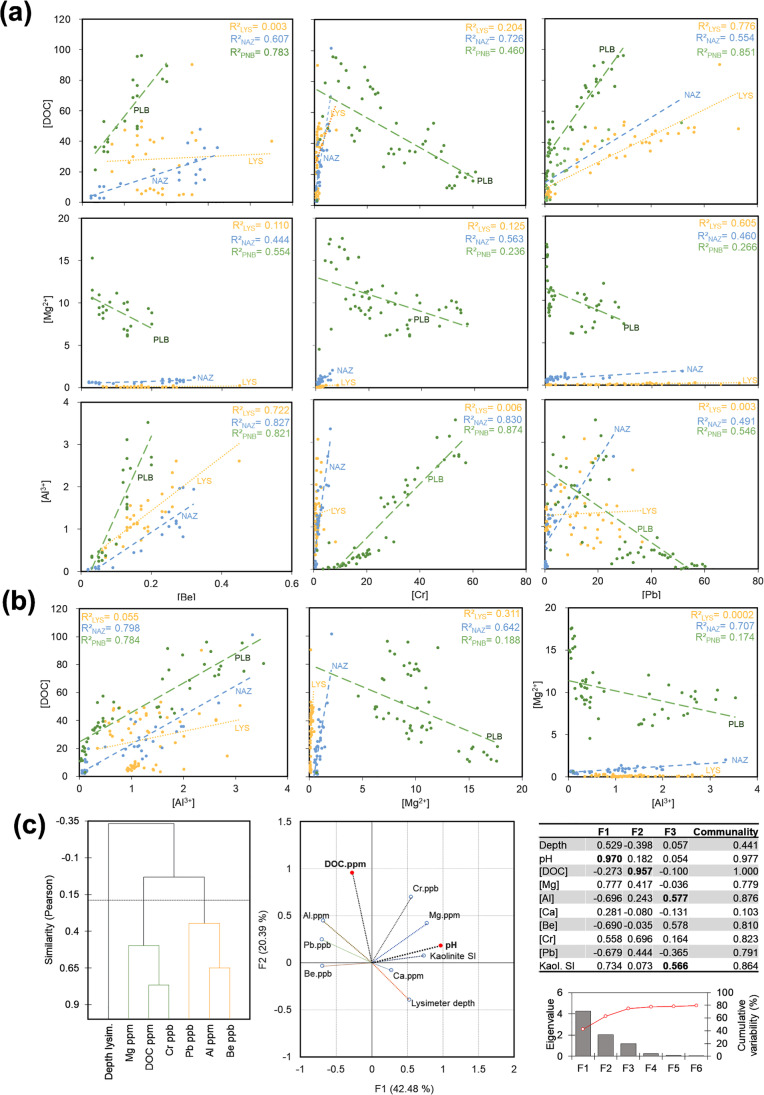
Linear correlations, agglomerative hierarchical clustering, and factor analyses. The leaching controls over dissolved concentrations of organic carbon, magnesium (Mg^2+^), and aluminum (Al^+3^) variable affect the ionic solution concentrations of Be, Cr, and Pb measured in the catchment areas under consideration (**a**). Potential autocorrelation effects in the different catchment areas for the leached major ions and DOC were evaluated (**b**). Results from AHC and factorial analysis (Pearson correlation, *α* = 0.25) corroborate soil pH, organic matter leaching, and regolith mineralogy are key master variables affecting Be, Cr, and Pb in soil solution collected at the researched catchment areas of the Slavkov Forest

The leaching, exchange, or retention of harmful metals in soils comprising our three catchments was previously thought largely dependent on soil pH and regolith mineralogy (e.g., Krám et al. 2017). Complex interactions among these parameters can inconsistently influence the measured concentrations of metal pollutants in our lysimeter-collected soil solutions, but some may linearly interact in a predictably way among them to account for up to 74.7% of the variability observed in the dataset (Fig. 3a–c, Supplementary Information ESM 1, Table ESM1). Together, they undoubtedly exert a major control over the chemistry of waters in transit at the vadose zone of the Slavkov Forest.

But the hydrochemistry of soil solution at different depths in the discrete catchments considered here may also vary as the result of other variables such as pore connectivity, or availability of negatively charged ligands capable of complexing ionic species from porewaters. Measurable soil features with the potential to influence these hidden control variables, responsible for ~ 25% of the variance we observed may include (i) soil structure, clay mineralogy, and texture and (ii) soil organic carbon (SOC) contents (Bache 1984; Rees et al. 1989; Evangelou 1998). Another external factor that may affect soil solutions would be direct absorption onto the nylon lysimeter samplers. But it has been determined that this factor has no effect on metal concentrations at pH < 5, i.e., LYS and NAZ, and would have only slightly decreased measured concentrations of elements such as Cr and Pb at pH ≥ 6 ( Wenzel et al. 1997; Martel et al. 2014)―i.e., only at the PLB site (Table 1).

Soil textures are similar among catchments and consist of loamy sand with low clay abundances. Clay effects over pore connectivity at the three catchments are thus similar and would have exerted no major measurable effect on the variability of our soil solution chemistries. X-ray diffraction of lower B horizons permitted identifying kaolinite as the sole weathering–induced clay mineral. Upon weathering, the parental granitic rock in LYS produces the highest subsoil kaolinite content (mean of 2.5 ± 0.5 wt. %) as an alteration product, with lower abundance at NAZ (< 0.5 wt.% kaolinite) and below this latter XRD quantification limit in PLB. The subsoils at these latter sites displayed a lack of Mg-bearing clay minerals, e.g., montmorillonite. From this observation, it was recently concluded that the weathering regime affecting (ultra)mafic-derived subsoil in NAZ (and by extension PLB) is unfavorable for Mg-rich clay authigenesis (Novak et al. 2020). Indeed, the mean chemical alteration index at LYS is 57% and in NAZ and PLB is 45 and 41%, respectively. These are characteristic values for fresh parental rocks (White and Buss 2003).

Regarding soil clay contents, a variable abundance of kaolinite and its metal reactivity (Miranda-Trevino and Coles 2003) might have variably affected our discrete soil solutions. This is because kaolinite may differentially immobilize ions with high affinity towards Al-oxyhydroxides out from the collected soil solutions, e.g., Be (Veselý et al. 2002). The reactive mineral surfaces of carbonates and oxyhydroxides would have exerted an important effect on metal immobilization in subsoil solutions from the NAZ and PLB sites. Under the strictly oxic conditions presumed at the subsoil levels in the low SOC catchments targeted here, pH acts as the master variable (Fig. 2). Therefore, differences in soil solution pH at the three sites mean that lysimeters that equilibrated at higher subsoil pH values would have collected samples likely affected by metal co-precipitation onto carbonate and oxyhydroxides formed at the sampler (Table 1). Within these samplers, Cr and Pb ions would have certainly reacted with Fe and Ca-Mg in (oxyhydr)oxides and carbonates, respectively (Fig. 2). In our case, this effect would have resulted in potential underestimation of total Cr and Pb soil solution contents in PLB and to lesser extent NAZ samples.

Regarding the immobilizing role of organic compounds in solution, in all catchments, the SOC contents were maximum at topsoil levels (A layer). Lower SOC levels existed at LYS, and the highest were at PLB topsoils. In all three sites, SOC decreased with increasing depth (Table 2). Given rather low SOC contents in the subsoils of the evaluated catchment areas, we inferred a minor SOC role in immobilizing ions leached and diffused out from the regolith―or diffused down after topsoil-runoff interactions. The SOC ligand effect would have been exclusively governed by subsoil pH. Subsoil pH at the LYS site means a diminished complexation capacity in all ligands comprising the SOC-derived compounds that account for the dissolved organic carbon (DOC) collected in our lysimeters. This DOC ultimately promote the formation of a slimy surface at the lysimeter sampler walls. The slimy sampler-wall surfaces would have harbored an abundance of metal-reactive organic ligands with pK_*a*_ values < 6 (e.g., carboxyl and amine functional groups). Such ligands included bacterial cells and their exopolymers that were likely fully protonated in LYS lysimeters, and, in consequence, had only a minor effect, if any, in decreasing our measured ionic soil solution concentrations. However, given higher soil pH values in the other studied sites (Table 2), deprotonated functional groups comprising the slimy sampler bottle surfaces would have immobilized an unquantified fraction of ions from soil solutions collected at the PLB and NAZ sites.

**Table 2:** Soil SOC contents, pH_H2O_, and base saturation

ID Site	Mineral soil (depth cm)	SOC (g kg^−1^)	Soil pH_H2O_	Lumped base Sat. (%)
LYS	(0–10)	26	3.8–4.3	7
	(10–20)	8.4		
	(20–40)	44		
	(40–90)	14		
NAZ	(0–10)	44	3.9–5.1	53
	(10–20)	19		
	(20–40)	5		
	(40–90)	2		
PLB	(0–10)	255	4.5–5.9	86
	(10–20)	31		
	(20–40)	14		
	(40–90)	-		

From the assessment of parameters capable of affecting the elemental concentrations of our pollution proxies, it is determined that for a range of metals, measurable soil solution concentrations may be diminished by complexation into organic and carbonate and oxyhydroxide phases formed or precipitated at the collector walls, particularly at sites featuring a circumneutral pH. An envisaged solution to that sampling issue is a sampling protocol modification that must contemplate collecting/substituting the lysimeter nylon bottles in the field, followed by digestion of the whole of their contents in the lab prior to elemental concentration analyses.

### Soil solution hydrochemistry

Zero-tension lysimeters allow for gauging the flux of metals associated with rainfall infiltration. They provide useful information to assess ecosystem input and output budgets. Tension lysimeters, on the other hand, allow comparing ion-exchange and/or uptake in relation to bedrock chemical weathering and elemental leaching (Goss et al. 2010; Martel et al. 2014; Makowski et al. 2020). With awareness of this sampling-target difference, and consideration of the hydrochemical effects of the soil parameters previously weighted, we consider now: (i) soil solution enrichments in Br, Cr, and Pb relative to regolith levels and (ii) the variability observed in the absolute concentrations. The observations are then used to interpret the extent to which the three proposed pollution-gauge elemental soil solutions concentrations reflect legacy pollution with noticeable changes triggered by seasonal runoff-shallow groundwater interactions occurring in the vadose zone. The data allow distinguishing inputs derived from concurrent atmospheric metal deposition at topsoil levels. Atmospheric metal deposition levels have been long-term monitored in mountainous forested catchment areas of the Czech Republic and are reported and evaluated elsewhere (see Oulehle et al. 2017, 2021).

#### Aluminum-normalized enrichments of soil solutions vs. regolith

**Fig. 4:**
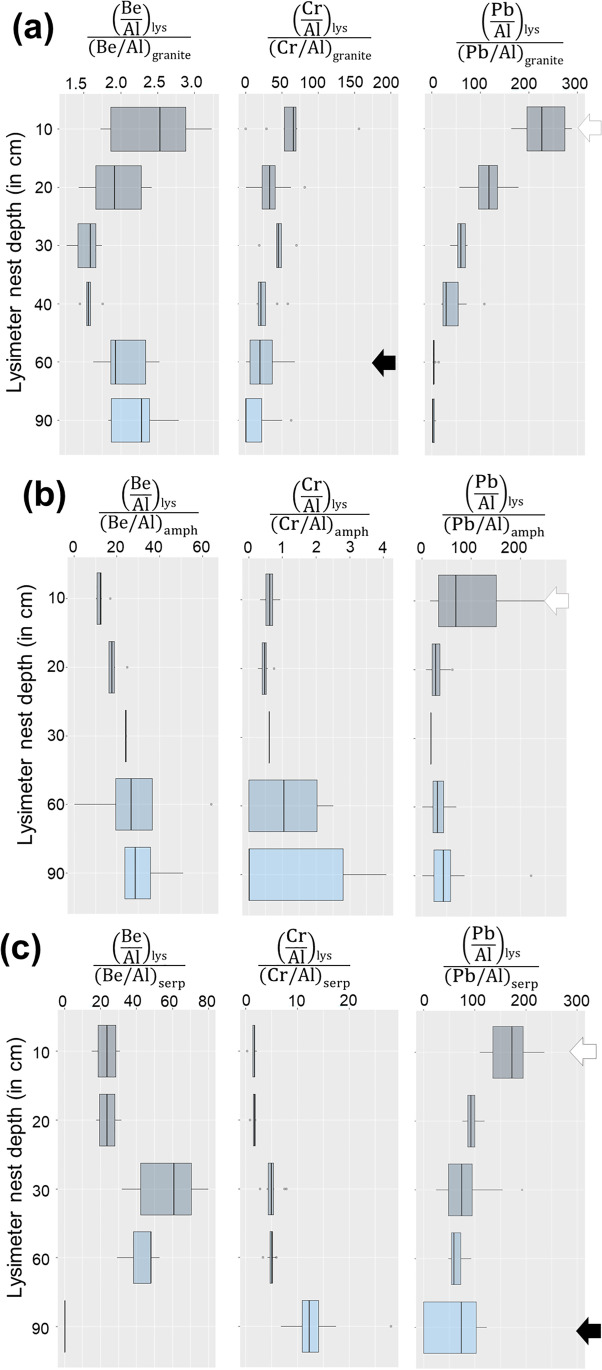
Box-plot of bedrock-normalized, soil depth statistical distribution of Be, Cr, and Pb concentrations in the three study sites, LYS (**a**), NAZ (**b**), and PLB (**c**), and during the 3-year monitoring period. This diagram shows relative enrichments of [Al]-normalized soil solutions at different depths and with regard to [Al]-normalized bedrock concentrations of the same elements

Soil solution distribution coefficients and their 3-year averaged changes with depth and site were calculated as the ratio of the aluminum-normalized soil solution averaged concentrations of Be, Cr, and Pb and the aluminum-normalized concentrations of the same elements in the regolith (Fig. 4). This data reduction approach reveals that in the felsic site (LYS), Be is enriched from 1.3 to 3.2 in the topsoil (0–40 cm depth) more probably because of regolith alteration. On the contrary, the enrichment in the subsoil levels (60 to 90 cm depth) ranged from 1.6 to 2.8. The granitic catchment LYS better portrays the significant atmospheric deposition of Cr in the Slavkov Forest. In LYS, this metal displays a topsoil enrichment that ranged between 13.6 and 156.1, while Cr in the subsoil solutions was up to 67.5 enriched compared to the regolith level (Fig. 4). This enrichment is considered the result of cumulative (legacy) pollution. Data resulting from this approach exposes also topsoil Pb enrichments of up to 278.7 and 235.6 at the NAZ and PLB sites, respectively, which corroborate that significant atmospheric heavy metal deposition occurred at the Slavkov Forest by the time of sampling.

Given high base-level Pb concentrations in the granite-derived soil, a coeval atmospheric Pb deposition at the LYS catchment was largely masked by runoff-shallow groundwater interactions with soil (in)organic ligands. A comparable situation occurs with Cr at the (ultra)mafic sites where relatively high base-level mask soil solution enrichments.

As with Cr in LYS, similarly, high Al-normalized Be and Pb were evident at the (ultra)mafic regolith-sourced soil solutions (Fig. 4). In NAZ, the 3-year-averaged distribution coefficients portrayed significant subsoil Be and Pb enrichments. A lower topsoil variability both at NAZ and LYS catchment signals a sufficiently long interaction between runoff and shallow groundwater bearing regolith-sourced legacy Be (and Pb). In PLB, the previous observation appears not valid as highly variable dissolved Be concentrations at about 30 cm depth occurred in parallel with low levels of this metal at the subsoil. This observation, and the fact that SOC are similar among catchments, and that more reactive clays are lacking in the basic regolith, is likely indicative of significant legacy Be complexed by reactive Fe-oxyhydroxides comprising alteration products at the (ultra)mafic-derived saprolite rocks. In consequence, distribution coefficient variability in our contrasting lithology sites provide insight into long-term ion-mineral (dis)equilibrium between the sampled solutions and the soil reactive components. Localized soil profile changes arise as a response to the physicochemical conditions governing metal complexation in the soil pore-water system.

#### Absolute seasonal Be, Cr, and Pb soil solution concentrations

**Fig. 5:**
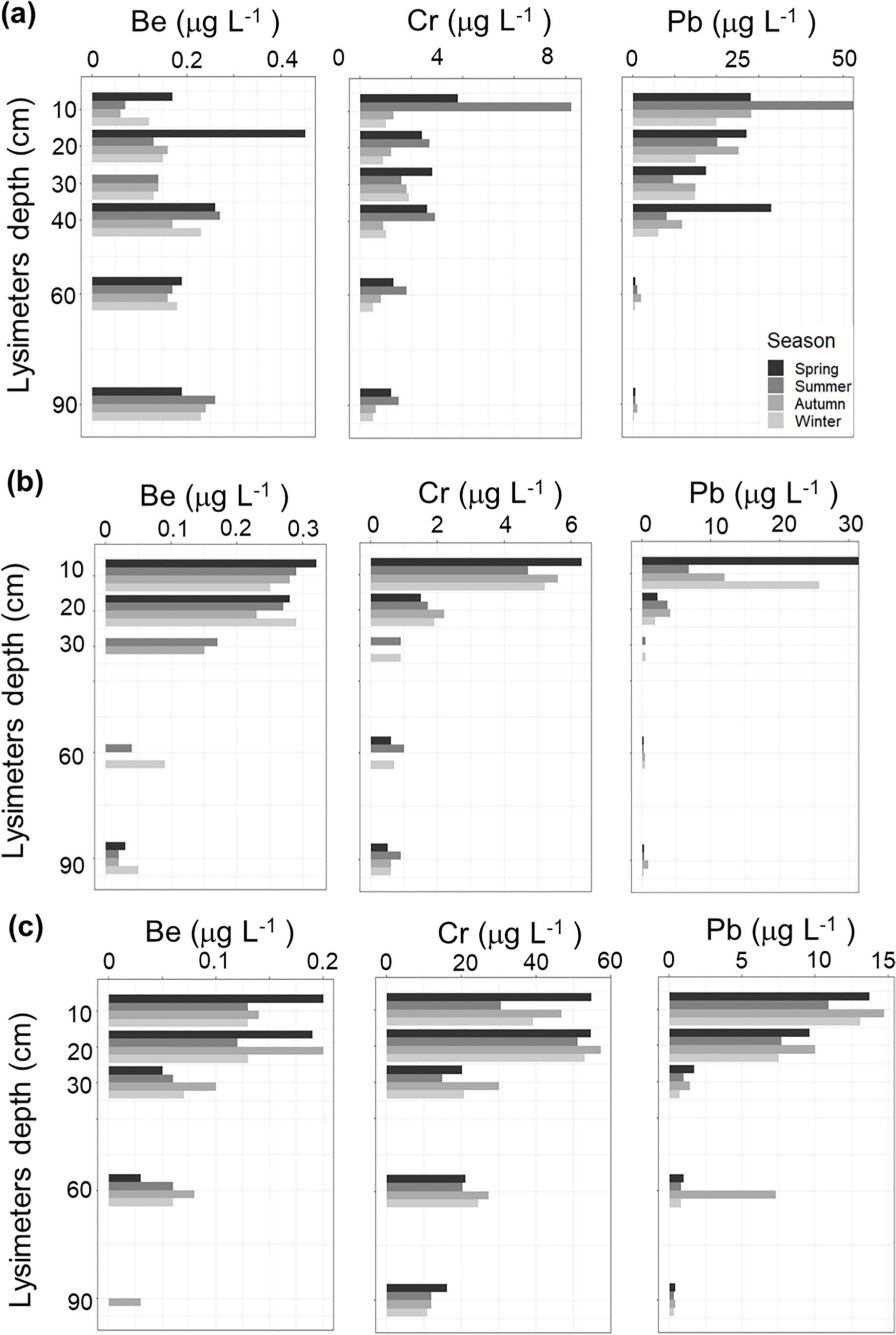
Seasonally discriminated depth profiles showing absolute measured soil solution concentrations. Supplementary Fig. 1 offers details on observed seasonal variations and their relation with collected soil solution volumes

The measured soil solution concentrations of Be, Cr, and Pb and their variability were seasonally discriminated. In this data treatment approach, each individual season in the 3-year dataset was considered a set of repeated observation for averaging seasonal concentration ranges at each site and depth within the soil profile (Fig. 5).

It emerges that the topsoil of the three sites received atmospheric metal deposition in excess to values measured in soil solutions collected in the subsoil and towards the regolith levels. However, we observed no clear seasonal single-element pattern that could be unambiguously attributed to atmospheric deposition. This is in conflict with observations made in other mid- to high-elevation forests of the Czech Republic where increased wet and dry airborne metal deposition during winter occurs in relation with the current E-W pollution gradient and frequent air-temperature inversions (Novák et al. 2005; Bohdalkova et al. 2012; Prechova et al. 2020; Buzek et al. 2023). Yet, the mobilization of metal pollutants could be linked to increased seasonality, with wet and dry periods marked by torrential rains and droughts that could significantly affect soil pore connectivity and biogeochemical responses (Blaurock et al. 2021; Patel et al. 2021). Therefore, we evaluated absolute seasonal Be, Cr, and Pb soil solution concentrations and considered runoff vs. groundwater contributions and residence times.

#### Time series concentration analysis

Time series concentration analysis revealed a more regular distribution pattern in the subsoil levels, but relatively high topsoil data dispersion. Soil solution volumes collected were also evaluated (Fig. 5, Supplementary Material ESM 2). A seasonal pattern is particularly evident at PLB where depth-related variability during the 3-year study period is high. Data showed that evapotranspiration may have exerted control over our measured subsoil Be, Cr, and Pb concentrations. An abrupt change in mean subsoil concentrations in excess to mean values measured at the topsoil levels appears to have followed a short-lived (2012) drought period (Supplementary Information ESM 2). Following this drought, increased pore connectivity at the topsoil level would have enhanced runoff-shallow groundwater interactions. During the following rainfall, such an enhancing effect resulted in evident changes in topsoil seasonal concentration values. The effect may have ultimately resulted in incomplete chemical equilibration with the variety of metal-reactive ligands in the waterlogged soil. The difference can be attributed to variable clay contents, but in the contrasting lithology catchment areas, temperature-dependent variations in evapotranspiration may have determined inversed concentration gradients at the discrete soil compartments evaluated.

### Runoff vs. groundwater contributions and residence times

Subsoil water has longer residence times than water in more mobile topsoil along the runoff pathways, and in the case of one of the studied catchment areas (LYS), it was previously estimated to be 13 to 18 months (Buzek et al. 1995). Similar values for the LYS catchment were obtained here, which validates our simplified approach. The corresponding mean residence times (MRT) for NAZ and PLB sites were between 6.6 and 10.3 months (Appendix). These transit time figures are longer than those observed in similar studies based on comparable soil types and at soil depths, ranging from 20 to 80 cm, where soil water MRTs were found to usually be less than 6 months (Lindström and Rodhe 1992; Dewalle et al. 1997). Longer MRTs could be linked to the proximity of the regolith at the catchment area examined here. Shallow groundwater transit times of hundreds of years have been determined deeper (> 15 m) in the critical zone (Ackerer et al. 2021).

## Summary and conclusions

Our combined dataset reflects the effects of atmospheric solutes deposited at the soil top levels and provide guidance for evaluating legacy inorganic chemical pollution in contrasting soil profile types. In summary:

(i) Rainfall collected in zero-tension lysimeters at topsoil does not equilibrate with subsoil solutions and clearly reflects atmospheric deposition of Cr and Pb. By the time of sampling, mid- to high-elevation Slavkov forest ecosystem received high wet and dry deposition of Be, and presumably other metals that were consistently collected by shallower zero-tension lysimeters.
(ii) Geochemical (dis)equilibrium between the soil and soil matrix water governed the hydrochemistry of the soil solutions at the time of collection, and complexation could have accounted for decreased concentrations with increased depths, particularly in sites exhibiting higher soil pH.
(iii) The complementary isotopic data constrain potential seasonal responses and point to sufficiently long water-saprolite interactions as to permit determining important contributions of shallow groundwater enriched in pollutants in our subsoil samples. As the mixed-source runoff waters are trapped in the lysimeter networks, deviations from the averaged-normalized concentration values hint at an effect exerted by seasonal water table recharge carrying legacy pollution.
(iv) At the watershed featuring a granitic bedrock, Pb is particularly enriched in topsoil solutions while, strikingly, Cr subsoil concentrations are within the same ranges measured at the topsoil (Fig. 5), which is seen as an indicative of legacy pollution. The resulting Cr concentration profile at this site may be due to decades of airborne transported Cr accumulated in the regolith and mobilized downward to the regolith. Yet, identifying legacy pollution in sites featuring (utlra)basic regolith by using solely the concentrations values of Cr in soil solution could be hindered by a much higher natural soil system abundances of reactive inorganic ligands derived from the weathering and alteration of the (ultra)mafic bedrock. The use of enrichment factors helps in the evaluation process.
(v) For sites featuring an underlying (ultra)basic bedrock lithology, the calculated Pb and Be enrichment factors revealed legacy pollution. This is reflected by higher seasonal variability at the bedrock-soil interface. The high variability of Be in topsoil at LYS contrasts with those determined in NAZ and PLB. Shallow groundwater-runoff mixing could therefore be of greater importance in the latter catchment areas.

Augmented concentrations of soil-leached heavy metals in runoff and shallow groundwater would likely ensue extreme weather events associated with the ongoing climate change. The polluted surficial water flows will first be discharged into mountainous water reservoirs, which would adversely affect their quality. Given the unprecedented need to build both resilience to climate change, and sustainable water access security, a plausible scenario of a seasonally enhanced leaching of latent soil metals requires us to adapt and/or improve existing land and reservoir management strategies. Overall, our study provides knowledge-based guidance for evaluating legacy pollution transport across soil profiles by using lysimeters placed in forested mountainous catchment areas of contrasting lithology.

### Electronic supplementary material

Below is the link to the electronic supplementary material.

Supplementary file1 (XLSX 580 KB)

Supplementary file2 Seasonal site-discriminated topsoil and subsoil collected soil solution Be, Cr and Pb concentrations. The lower panel shows the averaged-monthly volumes of soil solution collected.(EPS 1233 KB)

## Figures and Tables

**Fig. 6:**
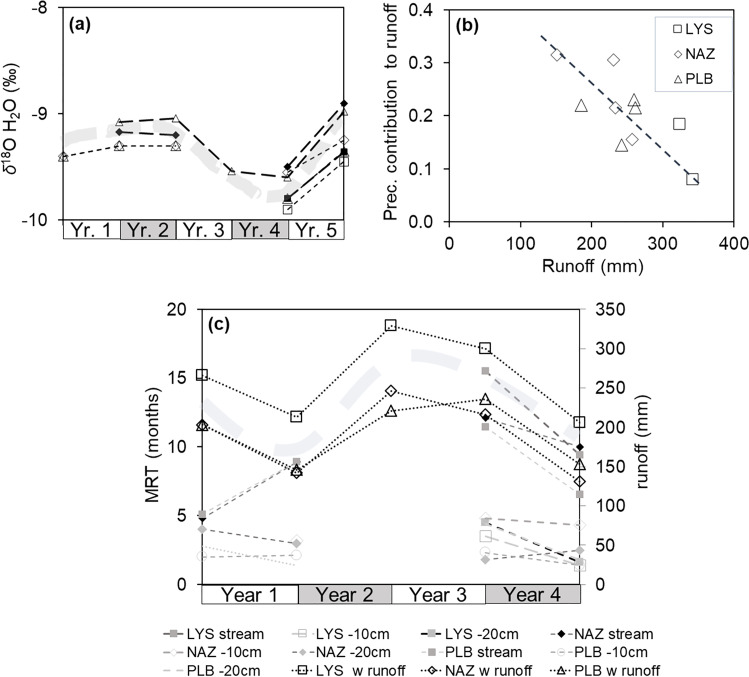
Selected runoff and water mixing stable *δ*^18^O isotope model outputs. Time-series of *δ*^18^O values in precipitation (•)―volume weighted mean, and stream water (○)―arithmetic all-year-average (**a**). A two-component runoff model for precipitation contribution to runoff vs. total runoff (**b**). A time-series plot for MRT estimation calculated from the *δ*^18^O precipitation values -axis and corresponding mean wintertime (November to April) runoff measurements in the secondary *y*-axis (**c**). Increasing runoff protracts MRT of precipitation in the catchments

## Data Availability

The entire dataset used is provided in Supplementary Information ESM 1: Tables ESM1 and ESM2. Reasonable requests for additional data would be addressed by the corresponding author.

## Appendix

### Stable δ^18^O runoff generation modelling

Information on mean residence times (MRT) of water in the unsaturated zone of soils is relevant for model validation. We estimated the residence times of runoff and the contribution of groundwater to runoff recharge in the studied catchment areas by implementing a stable *δ*^18^O isotope runoff water generation model (Buzek et al. 1995; McGuire and McDonnell 2006). Methodological details on this approach are provided below, in the “Runoff generation modelling approach” section.

The weighted seasonal average *δ*^18^O ratios and other parameters required for (and resulting from) our runoff generation modelling of a 3-year evaluation period are shown in Table

**Table 3:** Modelling parameters inputs and outputs from the runoff water generation model

Catch	Mean Q mm/month	MRT months	P_winter_	p_summer_	V_runoff_ mm	V_gw_ mm
LYS	24.3 ± 2.6	13.2 ± 2.3	0.13 ± 0.04	0.14 ± 0.04	399.8 ± 17.9	216.2 ± 20.6
NAZ	17.0 ± 1.5	8.9 ± 1.4	0.20 ± 0.03	0.31 ± 0.06	150.2 ± 29.6	115.4 ± 28.7
PLB	19.5 ± 1.3	8.0 ± 1.4	0.21 ± 0.01	0.25 ± 0.02	156.4 ± 31.3	126.3 ± 28.7

^3^ (see also Table ESM2 in Supplementary Information ESM 1 for details). Topsoil percolating soil waters were intermediate between rainfall isotopic composition (about − 9.4‰) and the less mobile “soil matrix water” sampled by tension lysimeters. This later water was slightly ^18^O-depleted (mean about − 11.2‰), pointing out to evapotranspiration effects (Fig. 6a). Therefore, seasonal evapotranspiration variations are thought attenuated in the *δ*^18^O values measured in runoff waters sampled by zero-tension lysimeters.

The season-averaged isotope ratios permit calculating runoff contribution. Accordingly, from Fig. 6b, it could be determined that direct runoff contributes about 27 ± 5% to stream discharge, Q (Table 3), and with the percent difference being attributed with ~ 72% certainty (i.e., *R*^2^ in the linear regression) to groundwater contribution. While in the evaluated period, the MRT of soil water may differ among watersheds (Fig. 6c, Table 3), they remain nearly constant throughout the evaluated period and appeared independent of variable volumes of runoff (Supplementary Information ESM 2).

The MRT of mobile water correlates linearly with runoff (Buzek et al., 1995). The estimated MRT can thus be used in combination with the parameter *p*, and runoff volumes to estimate with a high strength (*R*^2^ ≥ 0.9, α = 2.5), and by means of a negative linear relationship between groundwater volume and the *p* parameter, both the soil porewater and mobile water volumes can be determined. The intercept of that linear correlation is the mobile water volume, which for NAZ, as an example, yields 332 ± 21 mm (Fig. 6b). The difference with total water (i.e., V_runoff_ + V_gw_, Table 3) indicates that capillary soil porewater accounts for up 13% of the total volume. For LYS, this figure is 5.2%, and for PLB, it is 11.1%.

When compared using the volume of soil water, the estimated MRTs and soil porewater capacities point to soil saturation values between 50 and 60%. In consequence, at the topsoil, zero-tension lysimeters more probably collected significantly “new” rainfall in chemical disequilibrium with the soil system constituents, whereas tension lysimeters, by being located near our soil-regolith interfaces, collected waters that have long-interacted with reactive soil components. These waters included both capillary and water percolating the mineral soil. Due to their higher residence times, subsoil waters could partially be equilibrated with minerals undergoing alteration and weathering. Therefore, our subsoil samples recorded seasonal-induced variations in the dissolved shallow groundwater concentration of the three selected metal proxies.

### Runoff generation modelling approach

Stable oxygen isotope values (*δ*^18^O) of soil solutions were obtained by off-axis integrated cavity output spectroscopy (OA-ICOS, LGR3000, CA, USA). One µL of the sample was injected through a port heated to 80 °C. The vapor was transported into a pre-evacuated cavity and interrogated for its ^18^O/^16^O ratio relative to a standard (V-SMOW). The reproducibility of our *δ*^18^O_H2O_ determinations was better than 0.2‰. By combining our soil solutions *δ*^18^O_H2O_ values from tension lysimeters and those of runoff and precipitation, a two-component model of runoff generation was produced. The model is derived from the following general isotope mass balance (Eq. (1)):

1 $${\delta }^{18}{O}_{tot}=\frac{\sum {\delta }^{18}{{O}_{i}}^{*}{Q}_{i}}{{Q}_{tot}}\left[\permille\right]$$

where *i* are possible water sources, Q_*i*_ is its mass flow [m^3^], and *Q*_*tot*_ is the total flow [m^3^]. This mass balance can be used for discriminating event and pre-event components (Eq. (2)):

2 $${\delta }_{t}{Q}_{t}={\delta }_{p}{Q}_{p}+{\delta }_{e}{Q}_{e}\left[\permille.{\mathrm{m}}^{3}\bullet {\mathrm{s}}^{-1}\right]$$

where *Qt* is water flow [m^3^s^−1^]; *Q*_*p*_ and *Q*_*e*_ are contributing pre-event (groundwater) and event (rainfall, snowmelt) waters [m^3^s^−1^], respectively, and *δ*_*t*_, *δ*_*p*_, and *δ*_*e*_ are the corresponding isotopic compositions [‰]. Equation 2 can be solved parametrically to weigh the contribution of the event and pre-event (1 − *p*) waters (Eq. (3)):

3 $$p=\frac{{Q}_{e}}{{Q}_{t}}=\frac{{\delta }_{t}-{\delta }_{p}}{{\delta }_{e}-{\delta }_{p}}$$

Our mass balance is valid for any period of time, for example for winter or summer, if the mean seasonal isotope composition of all the components is known. The mean annual *δ*^18^O isotope composition (mean groundwater input), *δ*_*in*_, was estimated in our samples, and the mean *δ*^18^O_tot_ of the runoff water was determined.

A simple method for estimating the turnover time (i.e., mean water age) of the subsurface reservoir employs an exponential model for approximation. This estimation method is thought valid given that the distribution of transit times of water in the outflow has been though exponential, which corresponds to permeability decreases with depth within unconfined aquifers (Maloszewski and Zuber 1982; Buzek et al. 1991). In the case of stable oxygen isotopes, Siegenthaler (1979) demonstrated that the catchment water input (i.e., precipitation) can be approximated by a sinusoidal function with a 1-year period as per Eq. (4):

4 $${\delta }_{\mathrm{precip}}=\mathrm{D}+\mathrm{A}\; \mathrm{sin}\;\left(2 \delta \mathrm{t}\right)$$

where *D* is a constant, and *A* is the amplitude of *δ*^18^O variation in precipitation. Parameter *t* takes values between 0 and 1 for a full-year period. Under a simplifying assumption of constant infiltration and discharge, discharge can be similarly approximated by the factor B (Eq. (5)):

5 $$\delta_{\mathrm{discharge}}=\mathrm D+\mathrm B\;\sin\;\left(2\;\delta\mathrm t+\Delta\right)$$

where *B* is the amplitude of *δ*^18^O variation in output (discharge). Δ is the time shift of output variations in relation to the input. The mean transit time (T) in years can therefore be estimated (Buzek et al. 2006), e.g.:

6 $$\mathrm T=1/2\,\pi\,\left(\left(\mathrm B/\mathrm A\right)^{-2}-1\right)^{1/2}$$

A similar approach can be applied to soil solutions; *δ*_*precip*_ then represents the input, and infiltrated water values (*δ*_inf_) are used instead of *δ*_discharge_.
